# Subclinical Hypothyroidism in Geriatric Population and Its Association With Heart Failure

**DOI:** 10.7759/cureus.14296

**Published:** 2021-04-05

**Authors:** Priyanka Panday, Ana P Arcia Franchini, Beshoy Iskander, Fatima Anwer, Federico Oliveri, Fotios Kakargias, Pousette Hamid

**Affiliations:** 1 Research, California Institute of Behavioral Neurosciences & Psychology, Fairfield, USA; 2 Cardiology, California Institute of Behavioral Neurosciences & Psychology, Fairfield, USA; 3 Neurology, California Institute of Behavioral Neurosciences & Psychology, Fairfield, USA

**Keywords:** geriatric population, levothyroxine, subclinical hypothyroidism, thyroid dysfunction, heart failure, heart failure prognosis, elderly population

## Abstract

Heart failure (HF) is one of the most common causes of hospitalization in the geriatric age group, above 65 years. It is associated with high morbidity, mortality, and bad prognosis. Subclinical hypothyroidism (SCH) is a common condition present in this age group that significantly affects the cardiovascular system. Thus, this review attempts to elaborate on the association between subclinical hypothyroidism and heart failure in terms of their prevalence, pathogenesis, prognosis, and possible management in a geriatric age group. Among the various published literature on this topic on PubMed, PubMed Central, and Google Scholar, 36 relevant studies were selected to correlate this association. We found that both SCH and HF can be present concurrently in this age group. Especially in the geriatric population with thyroid-stimulating hormone (TSH) higher than ten mIu/L, there is an increased incidence of heart failure and a worse prognosis with preexisting heart failure. However, randomized controlled trials will be needed to explore further whether treatment is warranted or not in this age group.

## Introduction and background

Heart failure (HF) occurs when the ventricle cannot eject blood or fill blood. It is a common condition in the geriatric population and a prevalent cause for hospitalization in the geriatric population aged 65 and above [1]. Heart failure has a poor prognosis with more than 50% mortality within five years, and the mortality rate is also high [2]. It is a health care concern in the geriatric population with its significant morbidity and high cost associated with recurrent hospitalization [3].

**Figure 1:**
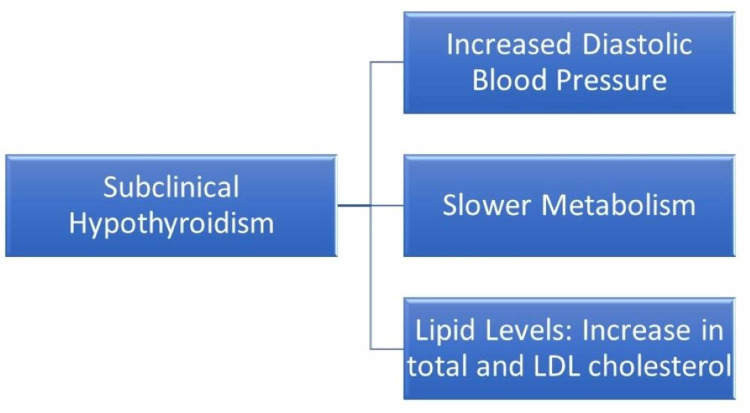
Common Effects of Subclinical Hypothyroidism LDL: low-density lipoprotein

Thyroid hormone has a significant impact on hemodynamic changes in the body. It has a substantial role in the heart's functioning and cardiovascular system-specifically, its vascular resistance, heart rate, and cardiac contraction actions [4]. Subclinical hypothyroidism (SCH) refers to increased thyroid-stimulating hormone (TSH) levels with normal free thyroxine concentrations. It is a common condition in the geriatric population with a prevalence of up to 18%. It is also associated with altered cardiac function [5,6]. Common known effects of subclinical hypothyroidism are represented in Figure 1.

It has been accounted that subclinical hypothyroidism is associated with atherosclerosis, heart failure advancement, and morbidity with cardiovascular diseases [4]. Impaired functioning of the systolic and diastolic heart has also been associated with subclinical hypothyroidism due to dysfunction of the vascular endothelium and vascular stiffness. This effect may eventually lead to heart failure [6]. Still, heart failure as a cardiac event is not much addressed in association with subclinical hypothyroidism in the geriatric population [5]. 

Despite the high prevalence of subclinical hypothyroidism, its treatment is reserved for patients at risk for overt hypothyroidism (TSH>10 miU/L), presence of antithyroid antibodies (TPOAb), infertile women, women of childbearing age, symptomatic and pregnant patients. There is very little evidence regarding the benefits and the risks of treatment in the geriatric age group [7]. In case, subclinical hypothyroidism is a risk factor or a cause for heart failure or associated with a worse prognosis of heart failure as comorbidity, it can be treated easily and prevented. Hence, the association is significant to be discovered [2,8].

A population-based study, healthy aging, and body composition study address the correlation of subclinical hypothyroidism with heart failure and its recurrence. This study consists of 2730 people of 70-79 years old with follow-up for four years to figure an association between TSH and cardiovascular impact. Comprised of 12.4% of the subclinical hypothyroid population, the outcome shows increased incidence and risk of recurrent heart failure about 3.26 times higher in the group with TSH>= 10. Whether subclinical hypothyroidism can cause or worsen existing heart failure is not clear [9].

A better understanding of why there is an increasing prevalence of subclinical hypothyroidism in aged people with heart failure and its effect upon heart failure prognosis is still unclear. Therefore, this literature review intends to clarify the correlation between these diseases in the geriatric population. It could also help resolve issues about the geriatric population's treatment with subclinical hypothyroidism in general and in the geriatric population with preexisting heart failure.

## Review

We reviewed scientific literature available on various databases PubMed, Cochrane, PubMed Central, Google Scholar with keywords and medical subject heading (MeSH) keywords. The search strategy for this literature review was done through keywords, as shown in Tables 1, 2.

**Table 1:** PubMed Search Through Keywords

Keywords and main keywords
Geriatric population, levothyroxine, subclinical hypothyroidism thyroid dysfunction, heart failure, heart failure prognosis
Hypothyroidism or subclinical hypothyroidism OR thyroid dysfunction: 64,351 results
Heart failure or left ventricular dysfunction or congestive heart failure: 304,787 results
Elderly or geriatrics or aged population :5,572,251 results
Main keyword: hypothyroidism or subclinical hypothyroidism or thyroid dysfunction and heart failure or left ventricular dysfunction or congestive heart failure and elderly or geriatrics or aged population: 856,004 results

**Table 2:** Search Through MeSH Keywords on PubMed MeSH: medical subject heading

MeSH keywords
(“Hypothyroidism/complications” {MeSH: NoExp} or “hypothyroidism/diagnosis” {MeSH: NoExp} or “hypothyroidism/drug therapy” {MeSH: NoExp} or “hypothyroidism/immunology” {MeSH: NoExp} or “hypothyroidism/mortality” {MeSH: NoExp} or “hypothyroidism/pathology” {MeSH: NoExp} or “hypothyroidism/physiology” {MeSH: NoExp} or “hypothyroidism/physiopathology” {MeSH: NoExp} or “hypothyroidism/therapy” {MeSH: NoExp}): 16632 results
(“Heart Failure/diagnosis” {MeSH: NoExp} or “heart failure/drug therapy” {MeSH: NoExp} or “heart failure/epidemiology” {MeSH: NoExp} or “heart failure/etiology” {MeSH: NoExp} or “heart failure/mortality” {MeSH: NoExp} or “heart failure/prevention and control” {MeSH: NoExp} or “heart failure/therapy” {MeSH: NoExp}): 81,446 results
Main keyword (combing mesh keywords and regular keywords): hypothyroidism or subclinical hypothyroidism or thyroid dysfunction and heart failure or left ventricular dysfunction or congestive heart failure and elderly or geriatrics or aged population and (“hypothyroidism/complications” {MeSH: NoExp} or “hypothyroidism/diagnosis” {MeSH: NoExp} or “hypothyroidism/drug therapy” {MeSH: NoExp} or “hypothyroidism/immunology” {MeSH: NoExp} or “hypothyroidism/mortality” {MeSH: NoExp} or “hypothyroidism/pathology” {MeSH: NoExp} or “hypothyroidism/physiology” {MeSH: NoExp} or “hypothyroidism/physiopathology” {MeSH: NoExp} or “hypothyroidism/therapy” {MeSH: NoExp}) and (“heart failure/diagnosis” {MeSH: NoExp} or “heart failure/drug therapy” {MeSH: NoExp} or “heart failure/epidemiology” {MeSH: NoExp} or “heart failure/etiology” {MeSH: NoExp} or “heart failure/mortality” {MeSH: NoExp} or “heart failure/prevention and control” {MeSH: NoExp} or “heart failure/therapy” {MeSH: NoExp}): 55 results

In the last 20 years, those articles published in the English language, including observational studies, controlled trials, clinical trials, meta-analysis, and review articles, were selected to get a clearer picture. Articles with animal studies, editorials were excluded from this study. After removing duplicates from the different databases and screening with relevance to our topic and criteria, a total of 55 articles are selected. Reviewing these articles, relevant 35 articles were chosen, and their findings were added to our paper.

Discussion

Effects of Subclinical Hypothyroidism in Heart 

TSH levels between 4.5 miU/L and 10 miU/L when T4 level is normal is defined as subclinical hypothyroidism [10]. Triiodothyronine T3, thyroxine T4 are hormones released by the thyroid gland. T3 is the active form that regulates the heart rate through its action on nuclear receptors on myocardial cells and can affect heart activity. It also acts on the vessel wall through smooth muscle cells via the transcription process [11,12]. Patients with Subclinical hypothyroidism are found to have cardiac function changes and endothelial activity. The lipid levels are altered, especially low-density lipoprotein, homocysteine, and blood pressure. These changes could lead to atherosclerosis earlier than other people [13,12]. An increase in vascular resistance due to subclinical hypothyroidism is believed to lead to diastolic dysfunction. However, all the studies do not confirm it [14]. Many meta-analyses have shown an increased risk of heart disease in the geriatric population with SCH but could not figure whether screening and treatment are valid or not [15]. In contrast, a study by Cappola et al. has shown subclinical hypothyroidism not to affect cardiovascular disease [10].

Heart Failure and Its Prevalence With Subclinical Hypothyroidism in Geriatric Population

The geriatric population encompasses most heart failure patients and that too with the worst prognosis [16]. There is a six to nine-fold greater risk of dying with sudden cardiac arrest among this age group than the average population [17]. With increasing age, the heart undergoes a variety of modifications. This process can lead to an increase in aortic pressure due to an increase in systolic blood pressure. This process of aging can lead to a stiff vessel wall. Muscle changes such as fibrosis increased muscle mass, and muscle cell loss is anticipated in the heart. These changes can lead to diastolic heart failure [13,17]. One study also showed that TSH increases in the geriatric population as they age and could be a part of aging than a pathology [18].

Various studies show that subclinical hypothyroidism is indeed associated with the development of heart failure and its worse outcome among heart failure patients. Another study Prospective Study of Pravastatin in the Elderly at Risk (PROSPER), a prospective cohort study was done among 70-82-year-old, 5316 total number with preexisting cardiac risk or disease for 3.2 years. This study revealed an increased incidence of heart failure present among those subclinical hypothyroid patients with TSH above ten compared to their euthyroid counterparts. It mentioned an increased hospitalization rate with HR of 4.99 (95% CI, 1.59-15.67) in patients with heart failure among patients with SCH [11,19]. A meta-analysis conducted by Ning et al. included 19,354 heart failure patients in total. It included 2173 patients with hypothyroidism as well. It established that SCH was indeed associated with worsening of heart failure. Thus, SCH leads to an increased risk of hospital admission [11]. Another meta-analysis with six large prospective studies with five studies in the geriatric population and including one of mean age 58 showed an increase in the risk of heart failure in both groups with high and low TSH, especially in a subgroup with TSH of more than 10 (hazard ratio {HR}: 1.86; CI: 1.27-2.72) [20].

Thyroid hormone replacement for subclinical hypothyroidism (TRUST) trial for subclinical hypothyroidism study reveals no significant change in the incidence of heart failure in a geriatric population (mean of 65 years) with SCH when started on thyroid hormone supplementation to the placebo group [18]. More than half of the geriatric population with heart failure have a minimum of two comorbidities that are not cardiac associated. For a long time, subclinical hypothyroidism can lead to heart failure even among those without any predisposing heart condition [21,11]. The prevalence of subclinical hypothyroidism among the geriatric population is high. When subclinical hypothyroidism is present with aging changes, it can increase heart failure progression [13]. Therefore, it is of utmost importance to manage the geriatric population's complex needs with heart failure by understanding its risk factors and precipitating causes [16]. The geriatric population with heart failure is not well represented in randomized controlled trials. As a result, there is a large gap in their proper management for their complex needs. Still, there is uncertainty in best addressing the complexities in treating HF [21,17].

How Can Subclinical Hypothyroidism Lead to Heart Failure?

Cardiac function, contraction, and relaxation can be affected by thyroid hormone imbalance and can lead to HF [18]. Few studies have mentioned that the thyroid activates SERCA2 encoded genes. SERCA2 system, Sarco/endoplasmic reticulum calcium ATPase isoform 2. This transporter is responsible for calcium reuptake into the endoplasmic reticulum from the plasma, and it determines the diastolic function of the heart. So, in cases where there is thyroid hormone deficiency, even relatively, it can affect the proper relaxation of ventricles in the diastole phase [12]. SCH is found to have caused dysfunction of diastole and systole at rest. These changes are supposedly reversible with euthyroidism status. Therefore there might be some beneficial effect on correction of TSH in terms of prognosis and heart function. However, a larger trial will need to be done [8,12]. A study by yin meng et al. included 75 patients with HF and SCH, which revealed there was an increase in the level of brain natriuretic peptide (BNP) with SCH, suggesting that SCH can be taken as a comorbidity of heart failure resulting in diastolic dysfunction, E/E′ ratio (the peak velocity of blood flow across the mitral valve in early diastole/the mitral annular velocity in early diastole ratio) was also found higher in SCH group in comparison to the euthyroid group [12]. Extracellular vesicles known as Apoptotic-derived microparticles can play a role in repairing endothelium and its injury. Few studies like that of Berezin et al. indicates that this EMP release is affected in patients with SCH and HF, contributing to endothelium dysfunction [18].

**Figure 2:**
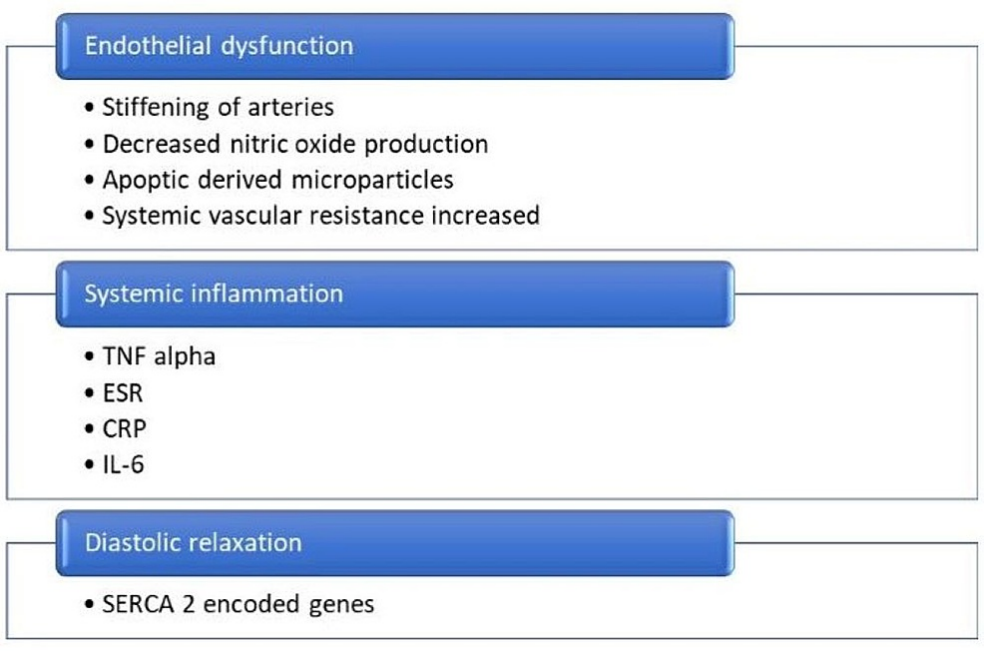
Pathogenesis of Subclinical Hypothyroidism and Heart Failure TNF alpha: tumor necrosis factor-alpha; ESR: erythrocyte sedimentation rate; CRP: C-reactive protein; IL-6: interleukin-6; SERCA 2: sarco/endoplasmic reticulum calcium ATPase isoform 2

Gupta et al. revealed a strong correlation between TSH and inflammation in patients with SCH. It suggests a role of the inflammatory markers like ESR, CRP, and interleukin-6. Therefore, inflammation can play a role in the heart's altered diastolic function with SCH [18]. Patients with preexisting HF cannot tolerate even slight thyroid hormone alteration [22]. A study by Owen et al. reveals that SCH causes increasing arteries' stiffening. Stiffening of arteries and dysfunctioning of cardiac diastole in old aged people are concerning factors for developing heart failure. SCH is associated with causing endothelial dysfunction through its action on the endothelium and alteration of nitrous oxide levels produced [18]. There is better heart functionality with the correction of thyroid status with shortened relaxation of the diastole phase, less ejection duration, and increment in ventricular ejection ratio while exercising [8]. Figure 2 summarizes the pathogenesis of subclinical hypothyroidism leading to heart failure.

Prognosis of HF With SCH 

Different studies performed epidemiologically have suggested lower thyroid hormone levels associated with increased risk of heart failure and bad prognosis [23]. A heart failure study with 1365 patients done by Kannan et al. with a mean around 57 years of age showed patients with SCH> 7 have more than three times the risk of mortality. They have an increased need for ventricular assist devices and heart transplantation, all leading to a bad prognosis [22]. The study by Muhammad et al. included 2335 patients with acute heart failure with a mean age of 65 admitted to the hospital. In terms of readmission in a month, patients with SCH had a higher risk for readmission in the heart failure with reduced ejection fraction group. Also, mortality from all causes was higher in SCH in the same group. So SCH could be independently used to predict one-month readmission in this group [24]. A study by Iervasi et al. with 3121 patients with a mean age of 61.1 years showed those with even TSH less than 10 showed an adverse prognosis between mortality from all-cause and cardiac death in hospitals admitted with acute heart failure [1]. 3121 cardiac patients with a subgroup of 208 subclinical hypothyroid with a mean age of 60.9 years were followed up for 32 months. It evaluated and showed an increased risk of death in these patients with preexisting heart conditions with even mild alteration in the thyroid level such as SCH [25]. Moon et al. did a study with analysis of a sub-group of 65 years with high cardiovascular risk, which included heart failure as a risk factor. Those with SCH had a high risk of mortality from all causes (relative risk {RR}: 1.41, CI: 1.08-1.85) [26].

What Are the Challenges of Treatment of Subclinical Hypothyroidism in the Geriatric Population?

American thyroid association recommends thyroid supplementation if TSH is more than 10, even dating back to 2005 [18]. It is considered beneficial to treat subclinical hypothyroidism in young patients, but much caution is taken regarding the geriatric population [27]. The medication used for SCH is levothyroxine sodium which has a narrow therapeutic index. It should be started in the lowest dose possible, especially in those with coronary heart disease history [19]. Thyroid hormone supplementation in the geriatric population a preexisting heart condition can lead to adverse effects as it can increase the body's oxygen requirement. The decision to treat the geriatric population for SCH is not taken as it is very challenging due to the adverse effects of hormone supplementation and unclear evidence of its benefits through RCT in this age group [15,13]. Somwary et al. pointed out that there is a significant risk of atrial fibrillation in the geriatric population if thyroid hormone supplementation is provided more than required. Therefore, it is of utmost importance in the geriatric population, to begin with, a lower dose in this age group. After that, observe patients closely and monitor their cardiac changes to increase the dose if needed [11].

Treatment is challenging, with some studies showing mortality increased with arrhythmia induced by treatment with levothyroxine in the geriatric population, especially those over 70. According to the European thyroid association, for this age group with TSH below 10 miU/l, treatment is not recommended with its risk of increasing cardiac ischemia and arrhythmia, especially in patients with heart disease [11]. In the geriatric population above 65 years, if TSH is more than 10 with symptoms and TPO positive, they are started on a low dose, whereas if TSH less than the long-term monitor is the best approach [28]. Risks of overtreatment with levothyroxine include bone loss, muscle loss, atrial fib, increased anxiety. Even after maintaining a stable dose, TSH needs to be monitored through TSH up to two times a year [29].

The Rationale for Treatment of SCH in HF Geriatric Population Patients

There is no clear understanding when it comes to treating patients with HF and SCH. As already known, thyroid replacement has a good effect on lipid profile, especially cholesterol and LDL, and maintaining blood pressure and carotid atherosclerosis [11]. Hayashi et al. state that worse cardiovascular outcomes could be predicted through SCH in patients hospitalized for acute HF. The European society of cardiology recommends assessing TSH in HF patients and acknowledges hypothyroidism's role in initiating acute HF. Still, there is no guideline about HF management with other TSH levels [18].

Thyroid hormone replacement in subclinical hypothyroidism improves the heart's diastolic and systolic function. The Cardiovascular health study by Rodondi et al. included 3044 people above 65 years of age who underwent one yr follow-up. Those with TSH over 10 had peak E velocity (95% CI, 1.09-1.18; P< 0.001) higher than others. Peak E velocity is a measurement that determines the heart's diastolic function while performing echocardiography. This measurement positively correlates with the incidence of heart failure. Simultaneously, the risk of heart failure was not raised in another elder group with TSH below 10. Therefore, it showed that the incidence of heart failure was significantly higher in the subthyroid geriatric population (HR: 1.88; CI: 1.05-3.34) in comparison to their euthyroid counterparts. This can be due to delayed relaxation of the left ventricle due to thyroid dysfunction. Cardiac function was noted over five years through echocardiography in about 70 percent of the group. Echo showed left ventricle mass to be increased in comparison to euthyroid. It is one of the changes that can be regressed with thyroxine therapy, and therefore levothyroxine supplementation reduces the risk of heart failure, preventing heart failure [11,18,30]. Rodondi et al. did a population-based study with 2730 people between 70 years and 79 years to figure an association between subclinical hypothyroidism and congestive heart failure (CHF) along with other cardiac problems. It included 338 SCH patients with four years follow-up period. Compared to euthyroid patients, CHF incidence was increased in the geriatric population with TSH 7 or higher, but no change in mortality was observed [8].

Few studies suggest levothyroxine supplementation be beneficial, particularly for heart failure patients in terms of the heart's functioning; given the prevalence of hypothyroidism in heart failure patients, supplementation is promising [23]. SCH treatment can improve lipid profile, but its benefit in cardiovascular risk or mortality from all causes is not clear to initiate treatment. The only condition considered is if there is a risk of converting to overt hypothyroidism. In that case, we can monitor thyroid levels. Treatment in overt hypothyroidism is essential as they are symptomatic and may develop myxedema. It is started with the lowest dose and monitored every six to eight weeks while increasing the dose slightly till hormone level is normal and the patients are yearly followed [31].

As a person ages, TSH increases and some study says it can lead to a long life. Nothing has been said about heart disease risk and mortality, especially in the preexisting heart failure population. RCT with older people is not enough to decide whether thyroxine supplementation in this age group is beneficial or instead reverting to euthyroidism can cause more harm [32]. Cardiovascular health studies including 679 SCH and 4184 euthyroid of at least 65 years were evaluated for 10-year risk of heart failure incidence. This study showed no correlation between heart failure or death from cardiovascular disease in the geriatric population due to SCH for a prolonged time. It was not significant at all [33]. Moreover, many studies show a risk of heart failure, and its prevalence is increased in SCH, usually for TSH more than ten, but no large RCT to prove if supplementation helps prevent HF [1].

Do We Need to Treat SCH in Geriatric Population Patients? 

TSH increases as age increases and can be a beneficial factor in longevity. Anything below 10 miU/L in most cases does not need medication unless thyroid antibodies, comorbidities are present, or pregnant women. Still, when it comes to TSH> 10 in the geriatric population, it can be considered for treatment after taking other factors into account like their comorbidities. There can be more harm than good, like fractures with their overtreatment risk [27]. A study showed that even with medication treatment, there was no improvement in various cardiovascular risk factors; therefore, it recommended no need to screen or treat the geriatric population patients with subclinical hypothyroidism [10]. Lily et al. did a study over four years in 65 years and older with 3996 patients with subclinical hypothyroidism to see its natural progression to over hypothyroidism. This study suggested that the groups with TSH 10 or higher would progress to overt nature, but more than half of people reverted to euthyroidism; it indicates that since most revert to euthyroidism, treatment might be futile [34]. A population-based study of 599 involving 85 to 89 years for 3.7 years in Leiden revealed decreased mortality rate with high TSH. This was suggested to result from slower metabolism resulting in decreased calorie intake by participants. The result was adjusted per other disability and health levels among participants [18].

A study with SCH women in the Netherlands showed the correlation between SCH and high risk for mi and aortic calcification. However, consensus regarding the long-term effect on the benefit of thyroid replacement in cardiac function improvement in SCH geriatric population patients lacks randomized controlled trials to form proper guidelines [11]. Even in the aged group, treatment with levothyroxine can be beneficial if their TSH is higher than ten or if they have antithyroid antibodies present or show symptoms. In these conditions, the chances of subclinical hypothyroidism persisting to overt hypothyroidism are high. So, medication can help combat hypothyroidism's adverse effects on the heart. Meanwhile, it is essential to be vigilant with the effects of medication clinically with treatment [19]. In contrast, another study suggested that even in the geriatric population, thyroid hormone supplementation can be safe if TSH levels are appropriately monitored and easily stopped if not beneficial [14].

Reference is still 4-5 miU/L, with age TSH increases, leading to more estimation of the geriatric population with SCH than prevalent in a geriatric population. There is a lack of large RCT that have shown promising effects from thyroxine replacement; the rationale is difficult. Treatment just based on anticipation of potential cardiovascular benefits does not seem realistic. Therefore, observation seems to be the best choice for this age group, unlike in young individuals [35].

The disparity can explain the overall disparity in different kinds of literature, trials, and the different result variation in the mean age of individuals, disparity of gender prevalence, comorbidities present, different follow-up duration, the difference with TSH levels present in SCH, and reversal of subclinical thyroid status to euthyroid during the study period in a geriatric population.

Limitations

Our study had many limitations. Firstly, our study was based on information collected through different databases, including different articles and not with direct patient contact. Secondly, it cannot be generalized to the younger group, and there is a lot of difference in the management of heart failure and subclinical thyroidism between these groups. Despite our limitations, our study shows that the geriatric population with TSH more than 10 has increased HF incidence, the prevalence of HF, and even those with preexisting HF have a poor prognosis. Therefore, it raises the question of whether the treatment of SCH in the geriatric population can improve HF outcomes.

## Conclusions

We addressed the relationship between subclinical hypothyroidism and heart failure in the geriatric population. We elaborated on the thyroid hormone's action on the cardiovascular system, emphasizing how subclinical hypothyroidism can lead to heart failure. Besides, we tried to rationalize thyroxine supplementation in elderly with heart failure among those with TSH greater than 10 miU/L. Though subclinical hypothyroidism can lead to diastolic dysfunction, it is still associated with many cardiovascular effects. When it comes to thyroxine supplementation in the elderly, this is debatable with a narrow therapeutic index of the treatment. Therefore, management regarding this condition is unclear. In the case of the elderly wait and watch approach is accepted generally. Even if thyroxine were to be given, it is started in the lowest dose possible with frequent follow-up, and close observation is recommended. Randomized controlled trials, placebo-controlled with a large number of the geriatric population, should be conducted to determine the clinical outcome for treating the geriatric population with subclinical hypothyroidism with heart failure. To solve the query of whether heart failure prevention or progression is possible with the treatment of SCH, it is must to conduct these trials.

## References

[1] Tognini S, Pasqualetti G, Calsolaro V, Polini A, Caraccio N, Monzani F (2014). Cardiovascular risk and quality of life in elderly people with mild thyroid hormone deficiency. Front Endocrinol (Lausanne).

[2] Yang G, Wang Y, Ma A, Wang T (2019). Subclinical thyroid dysfunction is associated with adverse prognosis in heart failure patients with reduced ejection fraction. BMC Cardiovasc Disord.

[3] Mizuno M, Kajimoto K, Sato N (2016). Clinical profile, management, and mortality in very-elderly patients hospitalized with acute decompensated heart failure: an analysis from the ATTEND registry. Eur J Intern Med.

[4] Sato Y, Yoshihisa A, Kimishima Y (2018). Subclinical hypothyroidism is associated with adverse prognosis in heart failure patients. Can J Cardiol.

[5] Nanchen D, Gussekloo J, Westendorp RG (2012). Subclinical thyroid dysfunction and the risk of heart failure in older persons at high cardiovascular risk. J Clin Endocrinol Metab.

[6] Baumgartner C, Blum MR, Rodondi N (2014). Subclinical hypothyroidism: summary of evidence in 2014. Swiss Med Wkly.

[7] Suh S, Kim DK (2015). Subclinical hypothyroidism and cardiovascular disease. Endocrinol Metab (Seoul).

[8] Rodondi N, Newman AB, Vittinghoff E, de Rekeneire N, Satterfield S, Harris TB, Bauer DC (2005). Subclinical hypothyroidism and the risk of heart failure, other cardiovascular events, and death. Arch Intern Med.

[9] Rodondi N, Bauer DC, Cappola AR (2008). Subclinical thyroid dysfunction, cardiac function, and the risk of heart failure. The cardiovascular health study. J Am Coll Cardiol.

[10] Cappola AR, Fried LP, Arnold AM (2006). Thyroid status, cardiovascular risk, and mortality in older adults. JAMA.

[11] Francois J, Al-Sadawi M, Casillas J (2020). Hypothyroidism and heart failure: epidemiology, pathogenetic mechanisms & therapeutic rationale. Int J Clin Res Trials.

[12] Meng Y, Zhao T, Zhang ZY, Zhang DK (2020). Association between sub-clinical hypothyroidism and heart failure with preserved ejection fraction. Chin Med J (Engl).

[13] Pasqualetti G, Tognini S, Polini A, Caraccio N, Monzani F (2013). Is subclinical hypothyroidism a cardiovascular risk factor in the elderly?. J Clin Endocrinol Metab.

[14] Monzani F, Dardano A, Caraccio N (2006). Does treating subclinical hypothyroidism improve markers of cardiovascular risk?. Treat Endocrinol.

[15] Mariotti S, Cambuli VM (2007). Cardiovascular risk in elderly hypothyroid patients. Thyroid.

[16] Díez-Villanueva P, Alfonso F (2016). Heart failure in the elderly. J Geriatr Cardiol.

[17] Lazzarini V, Mentz RJ, Fiuzat M, Metra M, O'Connor CM (2013). Heart failure in elderly patients: distinctive features and unresolved issues. Eur J Heart Fail.

[18] Bielecka-Dabrowa A, Godoy B, Suzuki T, Banach M, von Haehling S (2019). Subclinical hypothyroidism and the development of heart failure: an overview of risk and effects on cardiac function. Clin Res Cardiol.

[19] Hennessey JV, Espaillat R (2015). Diagnosis and management of subclinical hypothyroidism in elderly adults: a review of the literature. J Am Geriatr Soc.

[20] Gencer B, Collet TH, Virgini V (2012). Subclinical thyroid dysfunction and the risk of heart failure events: an individual participant data analysis from 6 prospective cohorts. Circulation.

[21] Azad N, Lemay G (2014). Management of chronic heart failure in the older population. J Geriatr Cardiol.

[22] Kannan L, Shaw PA, Morley MP (2018). Thyroid dysfunction in heart failure and cardiovascular outcomes. Circ Heart Fail.

[23] Vale C, Neves JS, von Hafe M, Borges-Canha M, Leite-Moreira A (2019). The role of thyroid hormones in heart failure. Cardiovasc Drugs Ther.

[24] Saad M, Lacoste AG, Balar P, Zhang A, Vittorio TJ (2020). The subclinical hypothyroid state might predict 30-day readmission in patients admitted with acute heart failure syndrome and reduced left ventricular ejection fraction. Ther Adv Cardiovasc Dis.

[25] Iervasi G, Molinaro S, Landi P (2007). Association between increased mortality and mild thyroid dysfunction in cardiac patients. Arch Intern Med.

[26] Moon S, Kim MJ, Yu JM, Yoo HJ, Park YJ (2018). Subclinical hypothyroidism and the risk of cardiovascular disease and all-cause mortality: a meta-analysis of prospective cohort studies. Thyroid.

[27] Calissendorff J, Falhammar H (2020). To treat or not to treat subclinical hypothyroidism, what is the evidence?. Medicina (Kaunas).

[28] Macedo Silva S, Carvalho A, Lopes-Pereira M, Fernandes V (2018). Subclinical hypothyroidism on the elderly. [Article in Portuguese]. Acta Med Port.

[29] Laurberg P, Andersen S, Bülow Pedersen I, Carlé A (2005). Hypothyroidism in the elderly: pathophysiology, diagnosis and treatment. Drugs Aging.

[30] Biondi B (2012). Mechanisms in endocrinology: heart failure and thyroid dysfunction. Eur J Endocrinol.

[31] Bensenor IM, Olmos RD, Lotufo PA (2012). Hypothyroidism in the elderly: diagnosis and management. Clin Interv Aging.

[32] Pasqualetti G, Tognini S, Polini A, Caraccio N, Monzani F (2013). Subclinical hypothyroidism and heart failure risk in older people. Endocr Metab Immune Disord Drug Targets.

[33] Hyland KA, Arnold AM, Lee JS, Cappola AR (2013). Persistent subclinical hypothyroidism and cardiovascular risk in the elderly: the cardiovascular health study. J Clin Endocrinol Metab.

[34] Somwaru LL, Rariy CM, Arnold AM, Cappola AR (2012). The natural history of subclinical hypothyroidism in the elderly: the cardiovascular health study. J Clin Endocrinol Metab.

[35] Biondi B, Cappola AR, Cooper DS (2019). Subclinical hypothyroidism: a review. JAMA.

